# Associations between the parent–child relationship and adolescent self‐worth: a genetically informed study of twin parents and their adolescent children

**DOI:** 10.1111/jcpp.12600

**Published:** 2016-07-18

**Authors:** Tom A. McAdams, Fruhling V. Rijsdijk, Jurgita Narusyte, Jody M. Ganiban, David Reiss, Erica Spotts, Jenae M. Neiderhiser, Paul Lichtenstein, Thalia C. Eley

**Affiliations:** ^1^ Institute of Psychiatry, Psychology & NeuroscienceMRC Social, Genetic & Developmental Psychiatry CentreKing's College LondonLondonUK; ^2^ Division of Insurance MedicineDepartment of Clinical NeuroscienceKarolinska InstitutetStockholmSweden; ^3^ Department of PsychologyGeorge Washington UniversityWashingtonDCUSA; ^4^ Yale Child Study CenterNew HavenCTUSA; ^5^ Office of Behavioral and Social Science ResearchNIHBethesdaMDUSA; ^6^ Department of PsychologyPennsylvania State UniversityPAUSA; ^7^ Department of Medical Epidemiology and BiostatisticsKarolinksa InstitutetStockholmSweden

**Keywords:** Adolescence, parenting, parent–child relationships, children‐of‐twins, self‐esteem

## Abstract

**Background:**

Low self‐worth during adolescence predicts a range of emotional and behavioural problems. As such, identifying potential sources of influence on self‐worth is important. Aspects of the parent–child relationship are often associated with adolescent self‐worth but to date it is unclear whether such associations may be attributable to familial confounding (e.g. genetic relatedness). We set out to clarify the nature of relationships between parental expressed affection and adolescent self‐worth, and parent–child closeness and adolescent self‐worth.

**Methods:**

We used data from the Twin and Offspring Study in Sweden, a children‐of‐twins sample comprising 909 adult twin pairs with adolescent children. Using these data we were able to apply structural equation models with which we could examine whether associations remained after accounting for genetic transmission.

**Results:**

Results demonstrated that parent–child closeness and parental‐expressed affection were both phenotypically associated with adolescent self‐worth. Associations could not be attributed to genetic relatedness between parent and child.

**Conclusions:**

Parent–child closeness and parental affection are associated with adolescent self‐worth above and beyond effects attributable to genetic relatedness. Data were cross‐sectional, so the direction of effects cannot be confirmed but findings support the notion that positive parent–child relationships increase adolescent self‐worth.

## Introduction

Self‐worth or self‐esteem has been defined as the level of regard that one has for the self as a person (Baumeister, Campbell, Krueger, & Vohs, 2003; Harter, 1993). High self‐worth indicates that a person has a positive view of her/himself, and that they believe that they are ‘good enough’. High levels of self‐worth are positively related to happiness (Baumeister et al., 2003), life satisfaction and well‐being (Proctor, Linley, & Maltby, 2009). Low self‐worth is associated with poor mental health, criminal behaviour, poor physical health, health compromising behaviours and poor economic prospects (Donnellan, Trzesniewski, Robins, Moffitt, & Caspi, 2005; McGee & Williams, 2000; Millings, Buck, Montgomery, Spears, & Stallard, 2012; Sowislo & Orth, 2013; Trzesniewski et al., 2006). Compelling evidence that low self‐worth *prospectively* predicts emotional problems comes from a recent meta‐analysis of 80 longitudinal data sets, collectively spanning childhood to old age, in which it was reported that low self‐worth predicted future depression and anxiety (Sowislo & Orth, 2013).

Evidence suggests that adolescence may be a developmental stage of particular importance to self‐worth and mental health, in that self‐worth is low for some individuals during this period (Birkeland, Melkevik, Holsen, & Wold, 2012; Block & Robins, 1993; Donnellan, Trzesniewski, Conger, & Conger, 2007; Hirsch & Dubois, 1991; Zimmerman, Copeland, Shope, & Dielman, 1997), and many common emotional and behavioural problems increase in prevalence and/or first manifest during adolescence (Ford, Goodman, & Meltzer, 2003). Researchers working on the large‐scale Dunedin birth cohort have shown that low adolescent self‐worth predicts a range of negative outcomes 10 years later in early adulthood, including depression, anxiety, nicotine dependence, criminal convictions and multiple physical health outcomes (Trzesniewski et al., 2006). This pattern of associations highlights the potential importance that adolescent self‐worth plays in development. Identifying factors that may have a positive influence on adolescent self‐worth may prove to be a crucial step in identifying how mental health might best be protected during this period of susceptibility.

### The parent–child relationship and self‐worth

Several studies have demonstrated that the parent–child relationship is linked to adolescent self‐worth, with close, affectionate, positive relationships associated with higher levels of self‐worth (Arbona & Power, 2003; Birkeland et al., 2012; Kernis, Brown, & Brody, 2000; Laible, Carlo, & Roesch, 2004; Parker & Benson, 2004). Longitudinal studies indicate that the parent–child relationship is predictive of *future* self‐worth during adolescence (Birkeland et al., 2012). This would suggest that positive relationships within the family may have the capacity to increase adolescent self‐worth, and thus improve mental health outcomes (Garber, Robinson, & Valentiner, 1997; Kamkar, Doyle, & Markiewicz, 2012).

To date, most studies examining associations between parent–child relationships and adolescent self‐worth have suffered from the same problems when it comes to the interpretation of findings – familial confounding. That is, parents and children are genetically related to one another, and share a family environment. Thus, if parental affection and offspring self‐worth are correlated, this *could* be indicative of social or environmental effects, but equally it could be attributable to familial confounding. Behavioural genetic studies demonstrate that when correlated phenotypes are both heritable, they often share genetic factors, both within individuals (Eley, 1997; McAdams, Gregory, & Eley, 2015; Michelini, Eley, Gregory, & McAdams, 2015; Plomin, DeFries, Knopik, & Neiderhiser, 2016) and across generations (D'Onofrio et al., 2003; Silberg, Maes, & Eaves, 2010, 2012). Twin studies show that self‐worth is 29%–62% heritable (Hur, McGue, & Iacono, 1998; Kendler, Gardner, & Prescott, 1998; McGuire, Neiderhiser, Reiss, Hetherington, & Plomin, 1994; McGuire et al., 1999; Neiss, Sedikides, & Stevenson, 2002; Raevuori et al., 2007; Roy, Neale, & Kendler, 1995). Similarly parental behaviour towards their children is under the influence of both children's genes (McAdams et al., 2013), and parental genes (Narusyte et al., 2008, 2011; Neiderhiser, Reiss, Lichtenstein, Spotts, & Ganiban, 2007; Neiderhiser et al., 2004). It could therefore be the case that genetic factors associated with a parent's capacity to have a close, affectionate relationship with their children, may be passed on to those children, in whom those genetic factors influence feelings of positive self‐worth. Thus, an important question to ask is whether associations between parenting and adolescent self‐worth remain after accounting for familial confounding.

There are several research designs capable of assessing genetic overlap between parenting and child traits. Child twin studies are the most commonly used, comprising twin children and their parent(s). Biometric analyses can reveal the extent to which child genetic factors involved in child behaviour correlates with those involved in parental behaviour. Where correlations are found, this indicates that children's genes involved in their own behaviour are also involved in evoking responses from their parents, an example of evocative gene–environment correlation (rGE). The presence of evocative rGE does not preclude the possibility that parent and child behaviour are influencing one another, but highlights that their aetiologies overlap. Child twin studies have demonstrated that associations between parenting and offspring phenotypes are frequently (at least in part) attributable to genetic overlap (McAdams et al., 2013; Reiss, Neiderhiser, Hetherington, & Plomin, 2000). To our knowledge, only one twin study has looked at the association between adolescent self‐worth and parenting. Reiss et al. (2000) examined the association between parental negativity and adolescent self‐worth in early and mid‐adolescence in a sample of adolescent twins, siblings and their parents. Results were mixed, showing that in early adolescence, the association between paternal negativity and adolescent self‐worth was partially attributable to genetic overlap. The association between maternal negativity and adolescent self‐worth was environmental in early adolescence but by midadolescence was largely attributable to genetic overlap (Reiss et al., 2000).

Child twin studies are limited because they measure only child genetic influences. Another design for assessing genetic overlap between parent and child traits involves the use of adult twin pairs with children (McAdams et al., 2014). The children‐of‐twins (CoT) design is a powerful method, capable of assessing whether intergenerational associations remain after accounting for the genetic relatedness between parent and child. This design has been used on a wide variety of parent–child associations (see McAdams et al., 2014 for a review), with some associations being attributable entirely or partially to genetic transmission (Silberg et al., 2010, 2012), and others remaining after accounting for relatedness (Eley et al., 2015; McAdams et al., 2015). Previously it has been reported that associations between parental depression and offspring self‐worth may be genetic in nature (Class et al., 2012). To date, no CoT studies have examined associations between the parent–child relationship and adolescent self‐worth.

### The present study

In our study, we used a genetically informed children‐of‐twins design to explore associations between adolescent self‐worth and two facets of the parent–child relationship: expressions of affection (parent behaviour) and reported closeness (parent feelings). We used adolescent‐reported self‐worth because we believe that adolescents are likely to have the greatest insight into their own subjective feelings of self‐worth. We used parent reports of parental affection and closeness so that the parent–child associations that we examined were not inflated by shared‐rater bias.

## Method

### Sample

Data were drawn from the Twin Offspring Study of Sweden (TOSS) and comprised 387 MZ and 489 DZ families. Families comprised a same‐sex twin pair (mother–mother or father–father), each with an adolescent child. Sample eligibility required that cousins (the offspring) were the same sex and could not differ in age by more than 4 years. Twins (parents) were 37% male, and 52% of offspring was male. Mean ages were 15.7 years for offspring (*SD* = 2.4; range 11–22), and 44.8 years for twins (*SD* = 4.9; range 32–60). After a complete description of the study was given, written informed consent was obtained. Further information on TOSS is given elsewhere (Neiderhiser & Lichtenstein, 2008).

### Measures

#### Closeness

The closeness within the parent–child relationship was assessed by parent report. Twins responded to 10 items assessing their perceptions of closeness within their relationship with their child (Hetherington, 1992; Reiss et al., 1995). Responses were on a 5‐point Likert scale ranging from ‘Not at all’ to ‘Very much’. Responses were scored such that higher scores indicate a closer relationship. Example items include ‘How close are you to the child?’. Cronbach's alpha was .81.

#### Expressions of affection

The directed expressions of affection towards the child by the parent were assessed by parent report. Twins responded to 22 items assessing the frequency with which they reported engaging in expressions of affection towards their child in the last month (Hetherington, 1992; Reiss et al., 1995). Responses were on a 7‐point Likert scale ranging from ‘Not at all’ to ‘more than once per day’. Responses were scored such that higher scores indicate more expressions of affection. Example items include ‘Told your child that you love him/her’. Cronbach's alpha was .88.

#### Adolescent self‐worth

This was measured via self‐report using a modified version of the Harter Perceived Competence Scale designed to measure self‐worth, self‐image and perceived competencies (Harter, 1982) in a Swedish population (Birgerstam‐Ouvinen, 1999; Ouvinen‐Birgerstam, 1985). The original scale comprised 72 statements relating to five subscales: (1) Physical appearance, (2) Skills and talents, (3) Physical well‐being, (4) Relations with family members and (5) Relations with others. To reduce conceptual overlap between our measures, we dropped the ‘relations with family members’ subscale. The final scale comprised 58 items. Responses were on a 4‐point Likert scale ranging from ‘Not at all right’ to ‘Exactly right’. Responses were scored such that a higher score indicates greater self‐worth. Cronbach's alpha was .91.

### Children‐of‐twins analyses

Using CoT data, it is possible to explore the aetiology of parent and child phenotypes, and the nature of intergenerational associations. The parent generation are twins, so variance in parent phenotypes can be decomposed into the effects of parental additive genetic (A1), common environmental (C1; nongenetic effects that make twins similar to one another) and nonshared environmental effects (E1; environmental effects that make twins different to one another). This is done by comparing the magnitude of MZ twin correlations (attributable to A1 + C1) to DZ twin correlations (attributable to (.5*A1) + C1). Comparing correlations between cousins from MZ and DZ families allows for the estimation of child genetic (A2) and nonshared environmental (E2) effects on child phenotypes. MZ cousins share 25% of their genetic variance, so correlations are attributable to .25*A2, whereas DZ cousin correlations are attributable to .125*A2. We do not calculate offspring shared environmental effects (C2) because including both C2 and C1 leads to an unidentified model.^1^ It is also worth noting that power is low to decompose variation in child phenotypes in CoT models, hence dropping C2 instead of C1. This low power arises because cousin correlations tend to be quite low, and because the relatedness coefficients of cousins (.25 and .125) are small (i.e. compared to twin relatedness coefficients of 1.00 and .50). This is not a major issue, however, as the focus of the CoT model is on the *intergenerational associations*.

Correlations between parent and child may involve genetic transmission (parent–child pairs share 50% of their DNA) and/or a phenotypic effect of parenting on child (or vice versa). Conversely, avuncular associations (associations between aunt/uncle and niece/nephew) cannot be attributed to a phenotypic effect (because aunts/uncles do not typically play a major role in child rearing) and so, if significant, indicate a role for familial effects. Because DZ twins share 50% of their genetic variance with their twin, they share 25% of their genetic variance with their twin's children. Similarly, as MZ twins share 100% of their genetic variance with their twin, they share 50% of their genetic variance with their twin's children. This means that the children of MZ twins are as related to their parent's co‐twin as they are to their own parent, but only their parent provides a rearing environment. Thus, if parent–child correlations are larger than MZ avuncular correlations, this suggests a role for exposure in explaining parent–child correlations. If MZ avuncular correlations are larger than DZ avuncular correlations, this suggests a role for genetic transmission. As such, comparing MZ and DZ avuncular correlations (relatedness coefficients of .50 and .25 respectively) and parent–child correlations allows for estimation of genetic transmission (A1′) versus direct ‘phenotypic transmission’: parent–child covariance not attributable solely to direct genetic transmission.

Prior to analyses, residuals were taken to control for twin sex and age, as is standard practice when working with twin data (McGue & Bouchard, 1984).^2^ We fitted structural equation models using maximum likelihood estimation in the programme OpenMx (Boker et al., 2011). The significance of pathways was tested by creating submodels in which paths were fixed to zero. Chi‐square difference tests were used to assess whether submodels yielded significantly worse fits to the data than the full model. The full children‐of‐twins model that we use, along with matrix specifications, has been published elsewhere (McAdams et al., 2015).

## Results

### Descriptive statistics

Descriptive statistics are reported in Table 1. After regressing on twin sex and age, all variables were close to normally distributed so we chose not to transform. Parent–child closeness and parental expressed affection were moderately correlated (*r *=* *.46, *p *<* *.001), indicating that the two constructs are related but distinct components of the parent–child relationship.

**Table 1:** Descriptive statistics

	Mean	*SD*	Range	Skew^a^	Kurtosis^a^
Adolescent self‐worth	26.85	4.53	16–46	−0.47 (−0.46)	3.31 (3.30)
Parent report parent–child closeness	39.09	4.66	21–50	−0.09 (−0.08)	2.75 (2.76)
Parent report expressions of affection	57.35	14.55	23–123	0.41 (0.45)	3.08 (3.34)

a skewness and kurtosis statistics refer to the distributions of the raw data, followed in parentheses by the distributions of the residuals of regressions on twin sex and age.

### Twin correlations

Intraclass correlations (estimated using maximum likelihood estimation in OpenMx) are given in Tables 2 and 3. Phenotypic associations (top row of Tables 2 and 3) were both significant. Twin (parent) correlations (second row of Tables 2 and 3) both followed the same pattern, with MZ correlations being larger than DZ, indicative of genetic influence. For parental affection, MZ and DZ correlations were significantly different (i.e. confidence intervals were nonoverlapping), indicating the presence of significant genetic effects. However, for parent–child closeness, MZ and DZ correlations were not significantly different to one another (confidence intervals overlapped). For both associations, parent–child correlations were significantly larger than MZ avuncular correlations, indicating that the phenotypic association was not entirely attributable to familial confounding. MZ and DZ avuncular correlations were very similar in magnitude and not significantly different from one another, indicating that genetic transmission does not play a role in intergenerational associations. Although point estimates indicated that cousin correlations were larger in MZ families than in DZ families (row 4 in Tables 2 and 3), overlapping confidence intervals indicate that we cannot interpret these differences as proof of heritability.

**Table 2:** Twin correlations (95% confidence intervals) between parental affection and adolescent self‐worth

	Parent‐reported affection and adolescent‐reported self‐worth	
	MZ	DZ
Parent–child correlation (affection and self‐worth)	.20 (.16, .25)	
Parental twin correlations (affection)	.51 (.43, .58)	.29 (.21, .37)
Avuncular correlations (affection and self‐worth)	.09 (.02, .16)	.08 (.02, .14)
Adolescent cousin correlations (self‐worth)	.14 (.04, .24)	.05 (−.04, .13)

**Table 3:** Twin correlations (95% confidence intervals) between parent–child closeness and adolescent self‐worth

	Parent‐reported parent–child closeness and adolescent‐reported self‐worth	
	MZ	DZ
Parent–child correlation (closeness and self‐worth)	.25 (.20, .29)	
Parental twin correlations (closeness)	.33 (.23, .42)	.25 (.17, .33)
Avuncular correlations (closeness and self‐worth)	.10 (.03, .18)	.12 (.06, .18)
Adolescent cousin correlations (self‐worth)	.14 (.03, .24)	.05 (−.04, .13)

### Structural equation modelling

**Figure 1:**
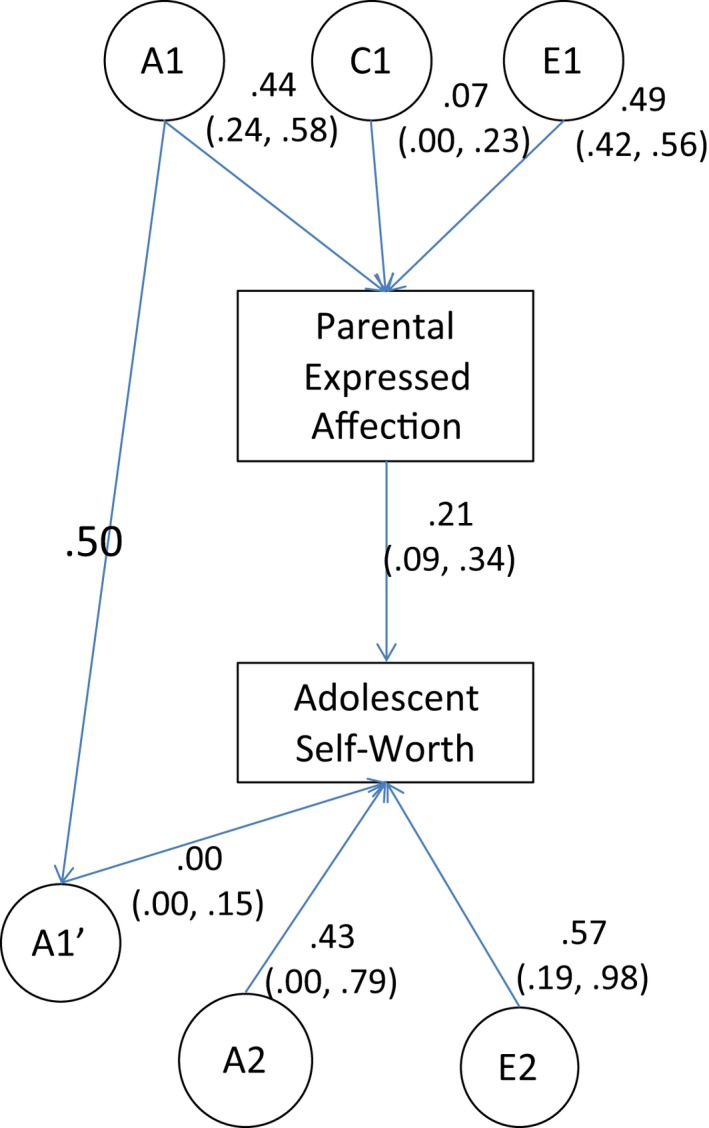
Structural equation models showing the association between parent‐reported affection within the parent–child relationship, and adolescent self‐worth. Path estimates are from the full (unconstrained) model in which all parameters are freely estimated. A1, Additive genetic effects on expressed affection; C1, shared environmental effects on expressed affection; E1, nonshared environmental effects on expressed affection; A1′, genetic effects common to parental expressed affection and adolescent self‐worth; A2, genetic effects specific to adolescent self‐worth; E2, nonshared environmental effects on adolescent self‐worth. The path between Parental Expressed Affection and Adolescent Self‐Worth is the phenotypic transmission pathway. NB, the pathway between A1 and A1′ is fixed to .50 because parents and children share 50% of their genome

**Figure 2:**
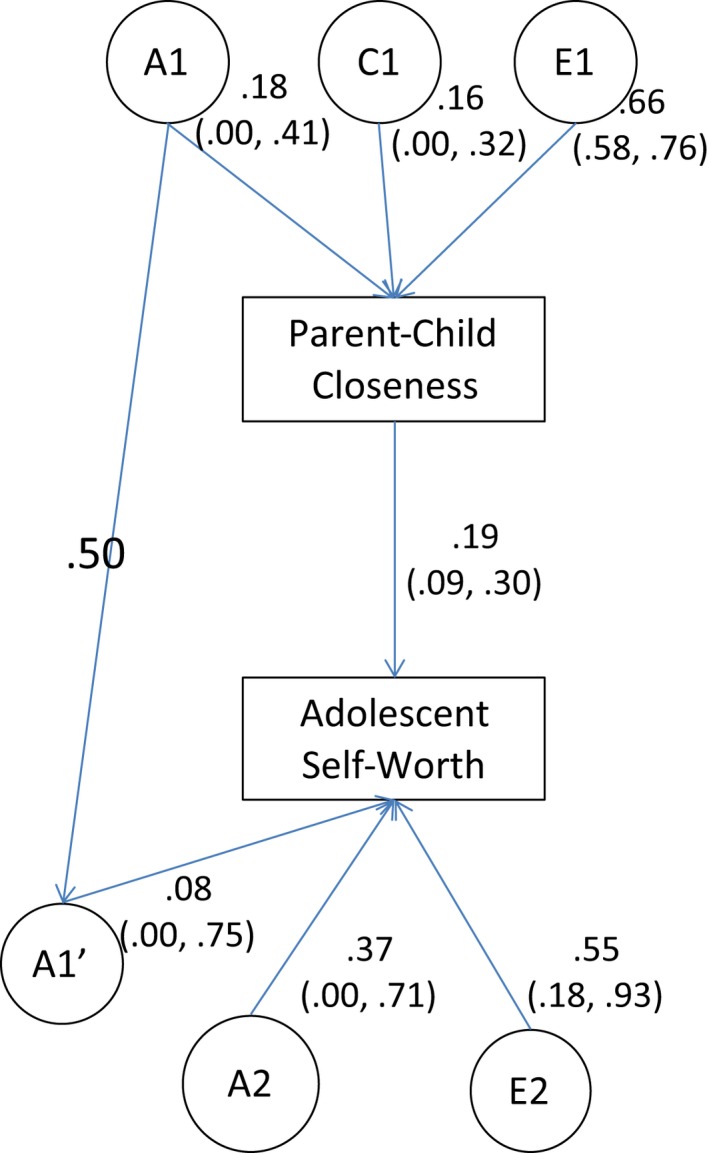
Structural equation models showing the association between parent‐reported parent–child closeness and adolescent self‐worth. Path estimates are from the full (unconstrained) model in which all parameters are freely estimated. A1, Additive genetic effects on closeness; C1, shared environmental effects on closeness; E1, nonshared environmental effects on closeness; A1′, genetic effects common to parent–child closeness and adolescent self‐worth; A2, genetic effects specific to adolescent self‐worth; E2, nonshared environmental effects on adolescent self‐worth. The path between Parent–Child Closeness and Adolescent Self‐Worth is the phenotypic transmission pathway. NB, the pathway between A1 and A1′ is fixed to .50 because parents and children share 50% of their genome

Models for one twin parent and their adolescent child are given in Figures 1 and 2. Models presented are unconstrained (i.e. no paths have been dropped). Submodels were fitted to the data, in which paths were dropped to test their significance. Model fit statistics are given in Tables 4 and 5. In both models, it was possible to drop the genetic transmission pathway (A1′ paths in Figures 1 and 2) without significantly changing model fit. However, dropping the phenotypic pathways (linking parenting with self‐worth) resulted in a significant loss of fit.

**Table 4:** Model fitting results for models including adolescent self‐report and expressions of affection in the parent–child relationship

	Parent‐reported parent–child affection and adolescent self‐worth				
	‐2LL	*df*	AIC	diff LL	*p*
Full model	9632.91	3,443	2746.91		
No A1′ pathway	9633.62	3,444	2745.62	0.71 (1)	.40
No Phenotypic pathway	9647.59	3,444	2759.59	14.68 (1)	.00

**Table 5:** Model fitting results for models including adolescent self‐worth and parent–child closeness

	Parent‐reported parent–child closeness and adolescent self‐worth				
	‐2LL	*df*	AIC	diff LL	*p*
Full model	9561.89	3,425	2711.89		
No A1′ pathway	9563.51	3,426	2711.51	1.62 (1)	.20
No Phenotypic pathway	9575.75	3,426	2723.75	13.87 (1)	.00

## Discussion

In the present study, we used a sample of twins with an adolescent child and found that associations between adolescent self‐worth and parent–child closeness/parental expressions of affection could not be attributed to genetic overlap. That is, the associations between the parent–child relationship and adolescent self‐worth involved *exposure*. Because we used cross‐sectional data, it was not possible to identify the direction of effects. The associations identified *could* constitute a positive effect of a close/affectionate parent–child relationship on adolescent self‐worth. Indeed, previous longitudinal research indicates that a positive parent–child relationship prospectively predicts adolescent self‐worth (Birkeland et al., 2012). Such an interpretation would align with research that emphasises the importance of parent–child relations to the emotional well‐being and development of the child. For example, parental warmth (which includes expressions of affection) is known to associate with adolescent mental health (Steinberg, 2001), and this may occur via promoting adolescent's feelings of self‐worth. Similarly, sharing a close relationship with a parent could have the same effect (Canetti, Bachar, Galili‐Weisstub, De‐Nour, & Shalev, 1997), and may also promote the ability of the adolescent to form bonds with others, which in turn could have positive consequences on their self‐worth (Wilkinson, 2004).

Alternatively, the direction of effects could be such that adolescents high in self‐worth have close and affectionate relationships with their parents. It may be easier for a parent to become close to a child with high self‐worth, and such a child may evoke more affection from their parents. Such an interpretation would align with the notion that adolescent behaviour can evoke responses from parents just as much/more so than parenting can influence offspring behaviour (Avinun & Knafo, 2014; Bell, 1968, 1979; Belsky, 1984; Fearon et al., 2015; Harold et al., 2013; Klahr & Burt, 2014). Many behavioural genetic studies highlight the ways in which an adolescent's genetically influenced behaviour (e.g. self‐worth) can evoke responses from the environment (in this instance, their parents).

Few genetically informed studies have examined parent–child associations involving either adolescent self‐worth or positive aspects of the parent–child relationship. Previous analysis of the TOSS sample has shown that the association between adolescent self‐worth and parental depression may be attributable to familial confounding, (Class et al., 2012). As such, ours is the first CoT study to report evidence for an association between parent behaviour and child self‐worth after accounting for familial confounds. Other CoT studies have examined associations between negative parenting behaviours and child outcomes, and our findings align with several of these studies in which associations survived correcting for familial confounding. Our examination of parent‐reported feelings of closeness within the parent–child relationship is the first CoT study to focus specifically on parental feelings (as opposed to behaviour), and suggests that the mechanisms underlying intergenerational associations may be similar to parenting behaviour.

The only child twin study to examine associations between parenting and self‐worth found that associations were often at least partially attributable to genetic overlap (Reiss et al., 2000). Methodological differences likely explain why Reiss et al. (2000) find genetic overlap where we do not: Child twin studies focus exclusively on child genes, so when Reiss et al. (2000) reported genetic overlap, the implication is that children's genetically influenced behaviour evokes maternal negativity. CoT studies can theoretically detect *both* parent and child genetic effects (although they have greatest power to detect parent genetic effects). Thus, the two types of data are used to ask different questions. Child twin models typically address variations on the question: to what extent do child genetic factors associated with child behaviour correlate with the parenting the child receives? However, CoT models address the question: after accounting for genetic relatedness between parent and child, do phenotypic associations between parenting and child behaviour remain? These questions are similar but distinct and we believe that CoT data provide the clearest method for accounting for familial confounds when studying intergenerational associations.

While the focus of our models was on intergenerational associations, we were also able to estimate aetiological influences on parenting and adolescent self‐worth. Parental‐expressed affection was found to be 44% heritable, similar in magnitude to most behavioural measures. Parent–child closeness, however, was not found to be heritable, with a nonsignificant estimate of 18%. Taken together these findings suggest that parent genetic factors play a smaller role in how close a parent feels to their child than in how affectionately they behave towards their child. In the current study, adolescent self‐worth was not found to be significantly heritable (although estimates were approximately 40%). As discussed in the method section of this manuscript, the use of cousin data to estimate aetiology results in low power to detect heritability, owing to the low relatedness coefficients used in calculations. As such, the lack of heritability in our study should not be taken as contradictory to previous studies that report significant heritability estimates for adolescent self‐worth.

### Strengths and weaknesses of the current study

The current study has several strengths. For example, different reporters were used for each of the phenotypes, so issues such as rater bias and shared method variance, which often artificially inflate associations, were not a problem. We were also able to explore associations using two measures tapping distinct aspects of the parent–child relationship (closeness and affection).

As with all studies, our findings should be considered within the context of certain limitations. For example, although we were able to control for familial confounding, it is still possible that unmeasured environmental variables could be confounding associations between parenting and adolescent self‐worth. It is also possible that parent/child gender may moderate the effects that we have examined. That is, the impact of paternal affection may be distinct from maternal affection, and boys’ self‐worth may be more/less affected by parental affection than girls’. Although we have not presented analyses by sex in the main text, we did explore sex differences and we include mean differences and twin correlations by parent and child sex in the supplementary materials available online (Tables S1–S9). While mean differences did exist, we found no compelling evidence that the nature of intergenerational associations varied by sex (hence we did not focus on sex differences in the main text).

The age range of our sample was wider than ideal (11–22), so the extent to which our results generalise to other adolescent populations is open to question. In follow‐up analyses, we found that although means varied with age, changes in the magnitude of associations between phenotypes were small, and there was no compelling evidence that the nature of intergenerational associations varied by age (see Tables S10–S13). Although it should be noted that our sample was underpowered to truly explore age and sex differences.

### Summary

The present study demonstrates that the association between the parent–adolescent relationship and adolescent self‐worth persists after accounting for familial confounds. While the direction of effects cannot be established in the current study, previous longitudinal research indicates that a positive parent–child relationship prospectively predicts adolescent self‐worth (Birkeland et al., 2012). If the direction of effects does run from parent–child relationship to adolescent self‐worth, then our findings would indicate that promoting a positive parent–child relationship could increase adolescent self‐worth, thereby promoting adolescent mental health in turn. Similarly, promoting adolescent self‐worth could have a positive impact on parent–adolescent relations.

**Key points**

- The parent–child relationship is often associated with adolescent self‐esteem and self‐worth.
- Associations between the parent–child relationship and adolescent self‐worth are difficult to interpret because parents and children share genes and a family environment.
- Using children‐of‐twins data, it is possible to test whether associations persist after accounting for relatedness.
- Parent‐reported parent–child closeness and parental expressions of affection towards their child both remain associated with adolescent self‐worth after accounting for familial confounding.

## Supporting information


**Table S1.** Means and standard deviations for all variables when splitting the sample into mothers and fathers.
**Table S2.** Twin correlations between parental affection and adolescent self‐worth for mothers and fathers separately.
**Table S3.** Twin correlations between parent–child closeness and adolescent self‐worth for mothers and fathers separately.
**Table S4.** Means and standard deviations for all variables when splitting the sample into male and female offspring.
**Table S5.** Twin correlations between parental affection and adolescent self‐worth for boys and girls separately.
**Table S6.** Twin correlations between parent–child closeness and adolescent self‐worth for boys and girls separately.
**Table S7.** Means and standard deviations for all variables when splitting the sample into male and female offspring and parents.
**Table S8.** Twin correlations between parental affection and adolescent self‐worth for mothers, fathers, girls and boys.
**Table S9.** Twin correlations between parent–child closeness and adolescent self‐worth for mothers, fathers, girls and boys.
**Table S10.** Mean differences by three age groups within the sample.
**Table S11**. Mean scores for two age groups (under 16; 16 and over).
**Table S12.** Twin correlations between parental affection and adolescent self‐worth for younger and older adolescents.
**Table S13.** Twin correlations between parent–child closeness and adolescent self‐worth for younger and older adolescents.

## Notes

1 When using covariance between cousins to examine the aetiology of a trait, it is also worth noting that the shared environmental component will refer not to the usual nuclear family environmental effects shared by siblings, but instead to the extended family environmental effects shared by cousins.

2 We also repeated analyses using residuals that controlled for twin *and* child sex and age. Results were very similar to those presented and conclusions do not differ dependent upon the residuals entered into structural equation models.
