# Supplementary material for: Associations between the parent–child relationship and adolescent self‐worth: a genetically informed study of twin parents and their adolescent children
Source: J Child Psychol Psychiatry. 2016 Jul 18;58(1):46–54. doi: 10.1111/jcpp.12600 (PMC5215430; doi:10.1111/jcpp.12600)
Supplement: Supplementary file 1 — Table S1. Means and standard deviations for all variables when splitting the sample into mothers and fathers. Table S2. Twin correlations between parental affection and adolescent self‐worth for mothers and fathers separately. Table S3. Twin correlations between parent–child closeness and adolescent self‐worth for mothers and fathers separately. Table S4. Means and standard deviations for all variables when splitting the sample into male and female offspring. Table S5. Twin correlations between parental affection and adolescent self‐worth for boys and girls separately. Table S6. Twin correlations between parent–child closeness and adolescent self‐worth for boys and girls separately. Table S7. Means and standard deviations for all variables when splitting the sample into male and female offspring and parents. Table S8. Twin correlations between parental affection and adolescent self‐worth for mothers, fathers, girls and boys. Table S9. Twin correlations between parent–child closeness and adolescent self‐worth for mothers, fathers, girls and boys. Table S10. Mean differences by three age groups within the sample. Table S11. Mean scores for two age groups (under 16; 16 and over). Table S12. Twin correlations between parental affection and adolescent self‐worth for younger and older adolescents. Table S13. Twin correlations between parent–child closeness and adolescent self‐worth for younger and older adolescents. [file JCPP-58-46-s001.docx]

Supporting information for ***Associations between the parent-child relationship and adolescent self-worth: a genetically informed study of twin parents and their adolescent children*** *by McAdams et al.*

**Follow-up analyses examining differences between mothers and fathers**

**Table S1.** Means and standard deviations for all variables when splitting the sample into mothers and fathers

|  | Male parent | Female parent | T test |
| --- | --- | --- | --- |
| Affection | 51.96 (13.70) | 60.52 (14.10) | -12.33, p<.001 |
| Closeness | 38.31 (4.66) | 39.54 (4.60) | -5.31, p<.001 |
| Self-worth | 38.61 (4.61) | 39.00 (4.51) | -1.73, p=.08 |

Results suggest that mothers report being more affectionate and feeling closer to their children.

**Table S2.** Twin correlations between parental affection and adolescent self worth for mothers and fathers separately

|  | Affection and self-worth | | | |
| --- | --- | --- | --- | --- |
|  | Dads | | Mums | |
|  | MZ | DZ | MZ | DZ |
| Parent-child | .25 (.18, .33) | | .16 (.10, .22) | |
| Parent | .50 (.35, .33) | .21 (.08, .33) | .46 (.35, .54) | .21 (.10, .32) |
| Avuncular | .16 (.02, .28) | .02 (-.07, .12) | .05(-.03, .14) | .10 (.02, .18) |
| Cousins | .18 (-.02, .35) | .00 (-.13, .13) | .11(-.01, .23) | .07 (-.04, .19) |

**Table S3.** Twin correlations between parent-child closeness and adolescent self worth for mothers and fathers separately

|  | Closeness and self-worth | | | |
| --- | --- | --- | --- | --- |
|  | Dads | | Mums | |
|  | MZ | DZ | MZ | DZ |
| Parent-child | .25 (.17, .32) | | .24 (.18, .30) | |
| Parent | .39 (.21, .53) | .18 (.04, .30) | .30 (.17, .41) | .29 (.18, .39) |
| Avuncular | .12 (-.02, .25) | .04 (-.05, .14) | .08 (.00, .17) | .17 (.09, .25) |
| Cousins | .17 (-.03, .35) | .00 (-.14, .13) | .11 (-.01, .23) | .08 (-.03, .19) |

The above intraclass correlations indicate that the association between paternal affection and offspring self-worth may be slightly stronger than that between maternal affection and self-worth, although confidence intervals overlap. Male avuncular correlations are stronger in MZ families than in DZ families, an observation suggestive of genetic transmission. This does not appear to be the case for maternal affection or closeness. It is worth noting however that confidence intervals are overlapping, indicating that differences are not significant.

**Follow-up analyses examining differences between boys and girls**

**Table S4.** Means and standard deviations for all variables when splitting the sample into male and female offspring

|  | Male adolescent | Female adolescent | T test |
| --- | --- | --- | --- |
| Affection | 56.46 (14.14) | 58.29 (14.91) | -2.62, p=.01 |
| Closeness | 38.97 (4.48) | 39.21 (4.84) | -1.03, p=.30 |
| Self-worth | 39.31 (4.32) | 38.37 (4.74) | 4.30, p<.001 |

Results suggest that girls receive more parental affection than boys, and boys report a greater sense of self-worth.

**Table S5.** Twin correlations between parental affection and adolescent self worth for boys and girls separately

|  | Affection and self-worth | | | |
| --- | --- | --- | --- | --- |
|  | Boys | | Girls | |
|  | MZ | DZ | MZ | DZ |
| Parent-child | .19 (.13, .26) | | .21 (.14, .27) | |
| Parent | .44 (.32, .54) | .19 (.07, .30) | .49 (.37, .59) | .22 (.10, .34) |
| Avuncular | .07 (-.03, .17) | .07 (-.01, .16) | .11 (.01, .21) | .07 (-.02, .16) |
| Cousins | .08 (-.06, .22) | .04 (-.08, .17) | .15 (-.00, .29) | .04 (-.09, .16) |

**Table S6.** Twin correlations between parent-child closeness and adolescent self worth for boys and girls separately

|  | Closeness and self-worth | | | |
| --- | --- | --- | --- | --- |
|  | Boys | | Girls | |
|  | MZ | DZ | MZ | DZ |
| Parent-child | .22 (.15, .28) | | .27 (.21, .33) | |
| Parent | .28 (.14, .40) | .18 (.05, .30) | .39 (.24, .52) | .29 (.18, .40) |
| Avuncular | .07 (-.04, .17) | .11 (.02, .20) | .12 (.00, .22) | .13 (.04, .21) |
| Cousins | .08 (-.06, .22) | .04 (-.08, .16) | .14 (-.01, .28) | .04 (-.09, .16) |

Results are not suggestive of any significant differences between boys and girls. For both, parent-child correlations are of a similar magnitude, but are larger than avuncular correlations. MZ and DZ avuncular correlations are similar in size for boys and girls. Self-worth may be more heritable in girls than boys (MZ cousin correlations for self-worth are slightly stronger in girls than boys).

**Follow-up analyses examining differences between mothers, fathers, girls and boys**

**Table S7.** Means and standard deviations for all variables when splitting the sample into male and female offspring and parents

|  | Boys | |  | Girls | |
| --- | --- | --- | --- | --- | --- |
|  | Dads | Mums |  | Dads | Mums |
| Affection | 52.32 (14.24) | 58.90 (13.52) |  | 51.57 (13.12) | 62.22 (14.49) |
| Closeness | 38.63 (4.56) | 39.17 (4.43) |  | 37.97 (4.75) | 39.93 (4.75) |
| Self-worth | 38.97 (4.37) | 39.51 (4.29) |  | 38.23 (4.82) | 38.46 (4.69) |

**Table S8.** Twin correlations between parental affection and adolescent self worth for mothers, fathers, girls and boys

|  | **Paternal** Affection and Adolescent Self-Worth | | | |
| --- | --- | --- | --- | --- |
|  | Boys | | Girls | |
|  | MZ | DZ | MZ | DZ |
| Parent-child | .31 (.21, .41) | | .19 (.08, .30) | |
| Parent | .50 (.26, .66) | .14 (-.04, .31) | .51 (.29, .67) | .30 (.12, .47) |
| Avuncular | .21 (.00, .38) | .00 (-.13, .13) | .11 (-.08, .27) | .05 (-.10, .18) |
| Cousins | .09 (-.20, .35) | .03 (-.15, .21) | .22 (-.05, .45) | -.03 (-.22, .16) |
|  | **Maternal** Affection and Adolescent Self-Worth | | | |
|  | Boys | | Girls | |
|  | MZ | DZ | MZ | DZ |
| Parent-child | .12 (.04, .21) | | .22 (.14, .30) | |
| Parent | .41 (.27, .53) | .23 (.07, .37) | .48 (.33, .60) | .18 (.02, .32) |
| Avuncular | .01 (-.10, .12) | .16 (.04, .21) | .12 (-.01, .24) | .08 (-.03, .20) |
| Cousins | .08 (-.08, .23) | .05 (-.11, .22) | .11 (-.08, .28) | .08 (-.08, .23) |

To explore the possibility that the mechanisms underlying the associations between closeness/affection and self-worth may differ for parent-dyad pairings of different gender, we ran ML correlational models on subsets of our sample. Results for parental affection show that for the fathers of boys, the parent-child association was .31. MZ avuncular correlations were noticeably larger than DZ avuncular correlations, but because of the wide and overlapping confidence intervals, these differences were not significant. (Wide confidence intervals and non-significant estimates are an issue throughout these parent-sex*child-sex analyses so we do not draw any firm conclusions but present them for reference.) For the fathers of girls, the parent-child association was .19. MZ and DZ avuncular correlations were not significantly different. For the mothers of boys the parent-child association was .12. MZ avuncular correlations were smaller than DZ avuncular correlations, but these differences were not significant. For the mothers of girls, the parent-child association was .22. MZ and DZ avuncular correlations were not significantly different to one another.

**Table S9.** Twin correlations between parent-child closeness and adolescent self worth for mothers, fathers, girls and boys

|  | **Paternal** Closeness and Adolescent Self-Worth | | | |
| --- | --- | --- | --- | --- |
|  | Boys | | Girls | |
|  | MZ | DZ | MZ | DZ |
| Parent-child | .20 (.09, .30) | | .29 (.18, .39) | |
| Parent | .33 (.07, .52) | .10 (-.11, .29) | .47 (.20, .64) | .23 (.05, .40) |
| Avuncular | .13 (-.08, .31) | .01 (-.12, .15) | .11 (-.10, .29) | .07 (-.07, .20) |
| Cousins | .08 (-.21, .35) | .04 (-.15, .21) | .22 (-.06, .45) | -.04 (-.24, .16) |
|  | **Maternal** Closeness and Adolescent Self-Worth | | | |
|  | Boys | | Girls | |
|  | MZ | DZ | MZ | DZ |
| Parent-child | .23 (.14, .30) | | .28 (.19, .35) | |
| Parent | .26 (.09, .40) | .24 (.08, .39) | .34 (.15, .50) | .30 (.15, .43) |
| Avuncular | .04 (-.08, .16) | .20 (.08, .31) | .14 (.00, .27) | .19 (.08, .30) |
| Cousins | .08 (-.08, .24) | .05 (-.12, .21) | .09 (-.09, .27) | .10 (-.06, .25) |

For paternal reports of closeness, patterns are very similar for boys and girls, with avuncular correlations all being non-significant. For maternal reports of closeness, Patterns are similar again, with point estimates indicating that DZ avuncular correlations are actually larger than MZ avuncular correlations, although differences are non-significant.

**Follow-up analyses examining age differences**

**Table S10.** Mean differences by 3 age groups within the sample

|  | Early | Mid | Late | Beta, p |
| --- | --- | --- | --- | --- |
| Affection | 64.90 (13.12) | 56.60 (13.33) | 49.17 (12.95) | -7.88, <.001 |
| Closeness | 39.89 (4.24) | 38.92 (4.699) | 38.32 (4.95) | -0.79, <.001 |
| Self-worth | 39.93 (4.57) | 38.26 (4.53) | 38.33 (4.33) | -0.83, <.001 |

Early adolescence: 11-14.5, N= 602

Mid adolescence: 14.5-17, N= 645

Late adolescence: 17-22, N= 498

**Table S11**. Mean scores for 2 age groups (under 16; 16 and over)

|  | Younger | Older | T test |
| --- | --- | --- | --- |
| Affection | 63.15 (13.38) | 51.45 (13.26) | 18.29, p<.001 |
| Closeness | 39.58 (4.40) | 38.58 (4.86) | 4.49, p<.001 |
| Self-worth | 39.45 (4.58) | 38.26 (4.45) | 5.50, p<.001 |

Younger; N= 879

Older; N= 866

**Table S12.** Twin correlations between parental affection and adolescent self worth for younger and older adolescents

|  | Affection and self-worth | | | |
| --- | --- | --- | --- | --- |
|  | Younger | | Older | |
|  | MZ | DZ | MZ | DZ |
| Parent-child | .24 (.16, .31) | | .13 (.05, .21) | |
| Parent | .41 (.25, .54) | .25 (.12, .37) | .39 (.23, .52) | .09 (-.06, .23) |
| Avuncular | .05 (-.08, .17) | .06 (-.05, .16) | .07 (-.06, .18) | -.03 (-.13, .08) |
| Cousins | .27 (.11, .41) | -.06 (-.20, .09) | .00 (-.17, .17) | .03 (-.12, .18) |

**Table S13.** Twin correlations between parent-child closeness and adolescent self worth for younger and older adolescents

|  | Closeness and self-worth | | | |
| --- | --- | --- | --- | --- |
|  | Younger | | Older | |
|  | MZ | DZ | MZ | DZ |
| Parent-child | .30 (.23, .37) | | .21 (.13, .29) | |
| Parent | .29 (.12, .43) | .20 (.05, .34) | .36 (.19, .50) | .33 (.19, .45) |
| Avuncular | .14 (.02, .26) | .13 (.02, .23) | .08 (-.05, .20) | .06 (-.04, .16) |
| Cousins | .28 (.12, .42) | -.06 (-.20, .09) | .00 (-.17, .17) | .03 (-.12, .18) |
